# An Investigation of Softening Laws and Fracture Toughness of Slag-Based Geopolymer Concrete and Mortar

**DOI:** 10.3390/ma13225200

**Published:** 2020-11-17

**Authors:** Yao Ding, Yu-Lei Bai, Jian-Guo Dai, Cai-Jun Shi

**Affiliations:** 1 College of Civil Engineering, Chongqing University, Chongqing 400044, China; dingyaohit@126.com; 2 Key Laboratory of Urban Security and Disaster Engineering of Ministry of Education, Beijing University of Technology, Beijing 100000, China; baiyulei@bjut.edu.cn; 3 Department of Civil and Environmental Engineering, The Hong Kong Polytechnic University, Hong Kong 999077, China; 4 College of Civil Engineering, Hunan University, Changsha 410000, China; cshi@hnu.edu.cn

**Keywords:** slag-based geopolymer, concrete, mortar, TPB test, softening law, fracture toughness

## Abstract

This paper aimed to determine the softening laws and fracture toughness of slag-based geopolymer (SG) concrete and mortar (SGC and SGM) as compared to those of Portland cement (PC) concrete and mortar (PCC and PCM). Using three-point bending (TPB) tests, the load vs. mid-span displacement, crack mouth opening displacement, and crack tip opening displacement curves (*P-d*, *P*-CMOD, and *P*-CTOD curves) were all recorded. Bilinear softening laws of the PC and SG series were determined by inverse analysis. Furthermore, the cohesive toughness was predicted using an analytical fracture model. The cohesive toughness obtained by experimental study was consistent with that predicted by analytical method, proving the correctness of the tension softening law obtained from inverse analysis. In addition, both initial and unstable fracture toughness values of SG mortar were lower than those of PC mortar given the same compressive strength. Moreover, the initial fracture toughness of SG concrete was generally lower than that of PC concrete, whereas the unstable fracture toughness exhibited an opposite trend.

## 1. Introduction

Slag-based geopolymer (SG) is an attractive alternative to Portland cement (PC). It can reuse the industrial by-product, i.e., ground granulated blast furnace slag (GGBFS), in an efficient way using alkali activation [1,2,3]. In addition, it reduces significantly the CO_2_ emissions produced during the production of PC, making it a greener solution [4,5]. The main hydration product of SG is C-S-H gel with a lower Ca/Si ratio than traditional PC and no zeolite or mica hydrations are found [1,2,3]. Extensive studies have demonstrated that SG can exhibit similar mechanical strength with or even perform better than PC does in many aspects, including low hydration heat, high early strength, good durability, and resistance to chemical attack [2,6]. However, several disadvantages such as quick setting, efflorescence, possibility of alkali–aggregate reaction, obvious shrinkage, and micro-cracks have also been stated [2]. In addition, SG exhibits a brittle behavior similar to that of PC.

As has been widely known, concrete is a quasi-brittle material and the dimension of its fracture process zone (FPZ) is comparable to the size of structure component. Hence, referring to this large nonlinear FPZ, it is improper to predict the failure process of a concrete specimen using linear elastic fracture mechanics (LEFM) [7,8]. Hillerborg et al. [9] then proposed the cohesive crack model to overcome the limitation of LEFM for concrete. The tension softening law, as a basic component of the cohesive crack model, is a material property representing the relationship between the crack opening displacement and the cohesive tensile stress along the crack. The softening law describes well the FPZ of concrete.

Fracture toughness is also a crucial parameter describing the fracture resistance ability of a material containing a crack. In general, concrete structures experience the following three stages during fracture process: under the external load, a crack first initiates and then propagates stably until an unstable fracture happens [10]. For the sake of obtaining the whole concrete fracture process, a double-*K* fracture criterion was proposed [10], which includes two size-independent fracture parameters, i.e., the initial fracture toughness $K_{Ic}^{ini}$ and the unstable fracture toughness $K_{Ic}^{un}$. The increase of fracture toughness from $K_{Ic}^{ini}$ to $K_{Ic}^{un}$ is attributed to the cohesive stress acting on the fictitious crack when the crack propagates stably, and can be defined as cohesive fracture toughness $K_{Ic}^{c}$ [11], which is relevant to the tension softening behavior of concrete. Moreover, the double-*K* fracture parameters can be calculated using an analytical method by conducting three-point bending (TPB) tests [11]. 

By far, very limited understanding has been achieved on the fracture properties (e.g., fracture toughness and tension softening law) of SG concrete and mortar. Ding et al. [12] compared the fracture energy (G*_F_*) and the characteristic length of SG concrete and mortar and PC concrete and mortar with comparable compressive strength, and found that the G*_F_* of SG concrete was always higher than that of PC concrete given similar compressive strength, whereas the G*_F_* of the mortar system exhibited an opposite tendency. However, obviously, these fracture properties are very essential for predicting the mechanical performance of SG structure elements subjected to static and dynamic load, and for achieving safe applications of SG materials [12,13,14,15,16,17,18,19,20]. Therefore, for this paper, the authors conducted a systematic experimental study to fill in the above gap. Three compressive strength levels varying from normal strength to high strength of PC and SG concrete and mortar were tested for comparison purposes. TPB tests were conducted according to the RILEM TC50-FMC [21] recommendation. The tension softening laws of PC and SG concrete and mortar were then determined using inverse analysis based on experimental results. Furthermore, the cohesive toughness of PC and SG concrete and mortar was predicted from the tension softening curves using analytical method and compared with the experimental values. The consistence of the cohesive toughness of PC and SG concrete and mortar between the experimental results and analytical ones validated the obtained softening laws and the double-*K* model. In addition, comparisons of tension softening curves and fracture toughness between PC concrete and mortar and their SG counterparts were discussed.

## 2. Materials and Methods

### 2.1. Raw Materials

The GGBFS used was from Nanjing, China. Table 1 lists the chemical composition of the GGBFS by mass, and its particle size distribution was primarily in the range of 0.4 μm to 100 μm.

The liquid alkali activator consisted of sodium silicate solution and sodium hydroxide. The water content and the modulus (the mole ratio of SiO_2_ to Na_2_O) of sodium silicate solution were 59% (by mass) and 3.7, respectively; and the sodium hydroxide (NaOH) flakes had a purity of 99%. A Grade 42.5 PC whose chemical composition is listed in Table 1 was adopted. The fine aggregate used was medium river sand with a fineness modulus of 2.47. The specific density and the water absorption of the fine aggregate were 2340 kg/m^3^ and 2.75%, respectively. Furthermore, gravel particles from a local river with a maximum size of 10 mm were selected as coarse aggregate. The bulk specific density and the water absorption of the coarse aggregate were 2530 kg/m^3^ and 1.83%, respectively.

### 2.2. Mix Proportions

Three compressive strength grades varying from 30 to 70 MPa were selected for both the PC and SG series. The mixtures of the PC and SG series listed in Table 2 and Table 3 are based on the former research conducted by the authors [12]. Powder polycarboxylate superplasticizer (SP) (BASF, Ludwigshafen, Germany) was used to assure the workability and the strength of the PC series. It is clear from Table 3 that the compressive strength of the SG series can be controlled by adjusting the amount of alkali concentration (*n*) and the modulus of the alkali activator (*M_s_*). All the concrete specimens had a constant sand ratio (SR) of 0.4, which was equal to the amount of fine aggregate per unit volume to that of the sum of fine aggregate and coarse aggregate.

### 2.3. Specimen Preparation

Both the SG and PC specimens were demolded 24 h after casting. All the specimens were cured in a curing chamber with a constant temperature of 21 ± 1 °C and a related humidity of 90% ± 5% for 28 days until testing. Three types of specimens were prepared for PC and SG concrete and mortar to conduct compressive tests, splitting tensile tests, and TPB tests, respectively. For compressive tests and splitting tensile tests, the mortar specimens had dimensions of 70.7 × 70.7 × 70.7 mm, while concrete specimens had dimensions of 150 × 150 × 150 mm. The dimensions of the mortar and concrete specimens were 100 × 100 × 515 mm when the TPB tests were conducted.

### 2.4. Testing Procedure

#### 2.4.1. Compressive and Splitting Tensile Strengths

A 2000 kN capacity universal testing machine was adopted to obtain the compressive and splitting tensile strengths of specimens. The loading rates operated were 0.5–1.0 MPa/s for the compressive tests and 0.05–0.10 MPa/s for the splitting tensile tests, respectively, according to the different compressive strength grades [22]. The splitting tensile strength *f*_t_ was calculated using the following equation:

(1) ft=2PπA=0.64PA

where *P* is the ultimate load (N) and *A* is the area of cross section (mm^2^).

#### 2.4.2. Three-Point Bending (TPB) Tests 

**Figure 1:**
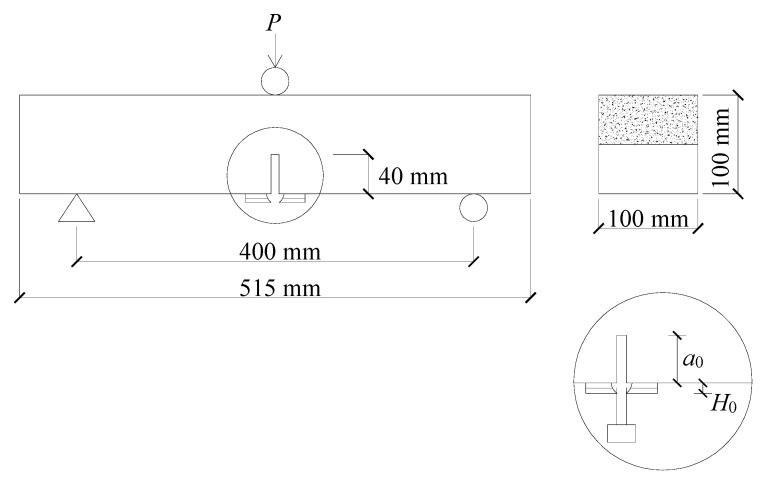
Configuration of the three-point bending (TPB) test beams [20].

The beams used for the TPB tests had a span/depth ratio of 4.0. All the specimens were pre-cut in the middle of the bottom side using a wet diamond saw, and the notch was 40 mm height and 3 mm wide (see Figure 1). A total of 48 beams were tested with four identical specimens in each group. Clip gauges were used to record the crack mouth opening displacement (CMOD) and the crack tip opening displacement (CTOD) of the beam. In addition, two high-precision displacement transducers (HPDTs) were employed to detect the mid-span displacement (*d*) of the beam. Furthermore, another two HPDTs were settled at both supports to eliminate their settlement influence on the mid-span displacement. A 200 kN capacity hydraulic jack was adopted to conduct the TPB tests. The machine was operated at a loading rate of 0.02 mm/min [23] in order to obtain the complete load vs. mid-span displacement (*P-d*), *P*-CMOD, and *P*-CTOD curves.

## 3. Experimental Results

**Figure 2:**
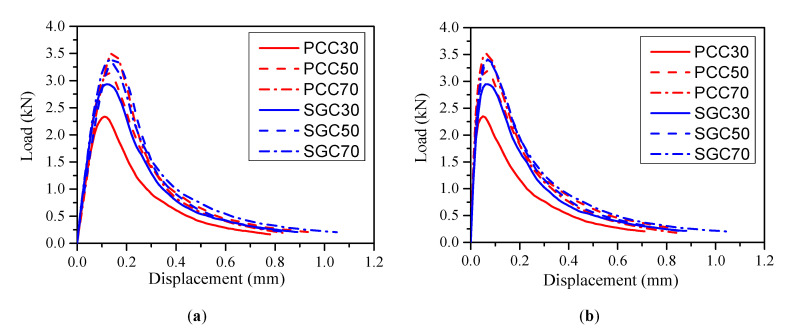
Typical (**a**) load vs. mid-span displacement (*P-d*) and (**b**) load vs. crack mouth opening displacement (*P*-CMOD) curves of Portland cement (PC) and slag-based geopolymer (SG) concrete beams.

Figure 2 presents the obtained average load–displacement (*P-d*) and *P*-CMOD curves of the PC and SG concrete beams in each group with different compressive strengths. The ultimate load *P*_u_, the initial cracking load *P*_ini_, the $CMOD_{c}$ and $CTOD_{c}$ at ultimate load *P*_u_, the modulus of elasticity *E* calculated from the *P*-CMOD curves [11], and the fracture energy G*_F_* calculated from the *P-d* curves [21] are summarized in Table 4, in which the average values of four identical specimens are provided. These parameters are also essential for calculating the fracture toughness in the following sessions. The initial cracking load *P*_ini_ was determined using a graphical method in this study, referring to the load value where non-linearity started on the *P-d* curves [11]. It can be concluded from Table 4 that the initial cracking loads of concrete were around 50–65% of their ultimate loads, whereas the initial cracking loads of mortar were approximately 80–95% of their ultimate loads. Concrete usually has a higher load resistance than mortar does after the crack is formed.

Table 4 indicates that the peak loads *P*_u_ of concrete and mortar beams increased with increasing compressive strength for both the SG and PC series as expected. The improvement of the average ultimate load *P*_u_ with the compressive strength increasing from C30 to C70 was more significant of the PCC beams than that of the SGC specimens. In the former case, *P*_u_ increased from 2.39 kN to 3.56 kN with a 49.6% increase, whereas only a 14.7% increase was observed in the latter case. Comparing the ultimate loads *P*_u_ between the PCC beams and the SGC beams, it is seen that, in the case of the C30 strength grade, the average ultimate load *P*_u_ of the PCC beams was 20.4% lower than its SGC counterpart. Nevertheless, with increasing compressive strength, the ultimate load *P*_u_ of the SGC beams became close to that of the PCC beams. It is known that the interfacial transition zones (ITZs) between the aggregates and the matrix are generally the weakest parts in low strength concrete. As a result, cracks prefer to occur in the ITZs and thus a stronger ITZ could lead to a higher ultimate load. The microscopic observations conducted by previous researchers [24,25] revealed that the ITZs between the SG paste and aggregates are denser and more homogenous than those between PC and aggregates. This explains the higher ultimate load of SG at C30. However, with a further increase of compressive strength, cracks may pass through the aggregates directly, so that the ITZ may exhibit comparable strength with the aggregate and trans-granular fracture happens. Moreover, the ultimate loads *P*_u_ of the PCM beams were always slightly higher than those of the SGM beams given the similar compressive strength grade. This could be explained by the high shrinkage of SGM [26,27,28], which would lead to more intrinsic micro-cracks and reduce the load-bearing capacity.

## 4. Determination of Softening Laws

The tension softening law is a basic component of the fictitious crack model (FCM) [9]. It is a material property representing the relationship between the cohesive stress and the crack opening deflection. The tension softening law is essential for predicting the fracture property of concrete, and can be obtained through direct tensile tests. However, the requirement for the direct tensile testing procedure is too restricted to meet [29,30,31]. Therefore, it is usually indirectly obtained by inverse analysis of TPB test results [6].

For practical applications, a bilinear strain-softening diagram is adopted by several researchers [29,30,32,33] and the CEB-FIP Model Code [34]. A general equation of the bilinear softening law is given by Equation (2):

(2) $$\left\{ \begin{array}{ll} \sigma =f_{t}-(f_{t}-\sigma_{s})w/w_{0} & 0\leq w\leq w_{s}\\ \sigma =\sigma_{s}(w_{0}-w)/(w_{0}-w_{s}) & w_{s}\leq w\leq w_{0}\\ \sigma =0 & w\geq w_{0} \end{array}\right.$$

The above equation includes four independent parameters, i.e., the tensile strength *f*_t_, the kink points ($\sigma_{s}$, $w_{s}$), and the crack width $w_{0}$ that corresponds to zero cohesive stress. Roelfstra and Wittmann [35] emphasized that for a simulation of the whole load–displacement curves of TPB tests, the most critical step is to determine the kink point of the bilinear softening law. 

The critical parameters of the bilinear strain-softening diagram can be determined by an inverse analysis indirectly [7,35,36] on the basis of the experimentally obtained load–displacement curves by conducting TPB tests. The inverse analysis adopts evolutionary algorithms, which is a biologically motivated iterative stochastic optimization method, and the core concept is to separate the variation of the object to be optimized from its evaluation as we find it in nature. During the iterative process, the assumed softening law is amended timely, so that the optimal match of the numerical data to the experimental outcomes can be obtained. The detailed calculation process, error definition, etc., can be found in [36]. The software CONSOFT [37] originally developed by Prof. Volker Slowik [37] and his colleagues at the University of Applied Sciences in Leipzig, Germany, was utilized to determine the softening laws of the PC and SG concrete and mortar. The program was based on the FCM [9], taking into account the boundary effect [38]. The boundary effect assumes that, within a transition ligament length at the end of the crack path, the local fracture energy decreases linearly, whereas, outside this district, the local fracture energy has a constant value.

The essential parameters of the bilinear softening laws of the PC and SG concrete and mortar obtained from the inverse analysis are summarized in Table 5. The values of $f_{t}$ used here were obtained from the splitting tensile tests. It is seen that for both the PC and SG concrete and mortar, the values of $w_{0}$ at the stress-free point and $w_{s}$ at the kink point decreased with increasing compressive strength. On the contrary, the values of $\sigma_{s}$ at the kink point mainly increased with increasing compressive strength. 

**Figure 3:**
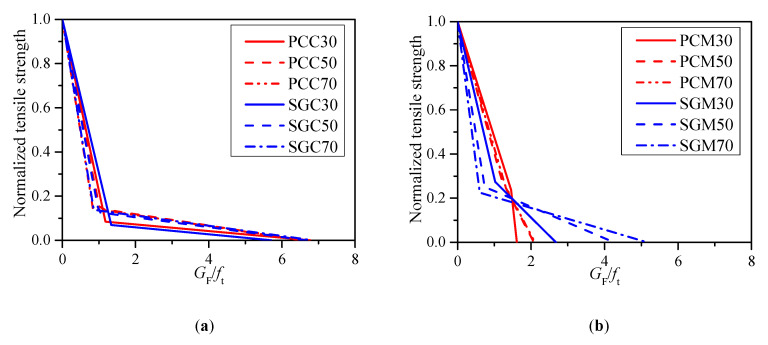
Bilinear softening laws of the PC and SG concrete and mortar. (**a**) PCC and SGC and (**b**) PCM and SGM.

Figure 3 shows the normalized bilinear softening curves of the PC and SG series with compressive strengths of 30, 50, and 70 MPa. Figure 3a shows that the normalized bilinear softening curves of PCC and SGC are generally the same given the same compressive strength, although the first descending part of SGC is slightly slower than that of PCC in the case of C30. Figure 3b clearly shows that that the first descending branches of PCM are usually gentler than those of SGM at all the three compressive strength levels. For both the SG and PC concrete and mortar, the first descending part becomes sharper with the strength increase.

**Figure 4:**
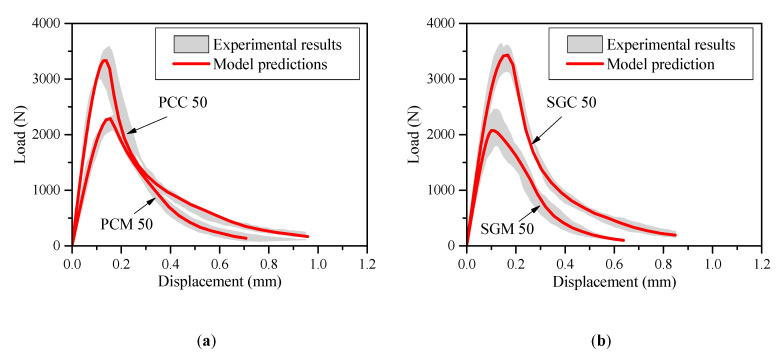
*P-d* curves obtained from experiment and prediction (**a**) PC mortar and concrete and (**b**) SG mortar and concrete.

With the obtained bilinear strain-softening diagrams, the load–displacement curves of the notched PC and SG concrete and mortar beams can be simulated [37]. Figure 4 presents the beams only with the compressive strength of 50 MPa for example. The shadowed areas represent the scatter of the experimental load–displacement curves of four identical specimens. It is clearly seen that the predicted *P-d* curves fit well with the experimental results, demonstrating the credibility of the bilinear softening laws obtained from backward analysis. 

## 5. Determination of Fracture Toughness

### 5.1. Experimental Approach

Xu and Reinhardt [10,11,39] proposed the double-*K* fracture criterion, including the initial toughness $K_{Ic}^{ini}$ and the unstable toughness $K_{Ic}^{un}$, to judge the fracture characteristics of cementitious materials. The initial toughness $K_{Ic}^{ini}$ corresponds to the initial stress intensity factor created by the initial cracking load $P_{\mathrm{ini}}$ that can be calculated by Equation (3), where $a_{0}$ is the depth of the pre-cut notch and defined as the initial crack length [11]. $K_{Ic}^{un}$ corresponds to the critical stress intensity factor generated by the maximum load that can be calculated by Equation (4), where $a_{c}$ refers to the critical crack length that can be evaluated by Equation (7) [11].

(3) KIcini=3PiniSa02H2BF(a0H)

(4) KIcun=3PuSac2H2BF1(acH)

(5) F(a0H)=1.99−(a0H)(1−a0H)[2.15−3.93(a0H)+2.7(a0H)2](1+2(a0H))(1−a0H)1.5

(6) F1(acH)=1.99−(acH)(1−acH)[2.15−3.93(acH)+2.7(acH)2](1+2(acH))(1−acH)1.5

(7) $$a_{c}=\frac{2}{\pi }\left(H+H_{0}\right)\arctan\sqrt{\frac{BE}{32.6P_{u}}CMOD_{c}-0.1135}-H_{0}$$

(8) $$E=\frac{6Sa_{0}}{C_{i}BH^{2}}\left(0.76-2.28\alpha_{0}+3.87\alpha_{0}^{2}-2.04\alpha_{0}^{3}+\frac{0.66}{{\left(1-\alpha_{0}\right)}^{2}}\right)$$

where B, H, and S are the breadth, height, and span, respectively, of the TPB beam; $CMOD_{c}$ is the critical crack mouth opening displacement; $H_{0}$ is the thickness of the clip gauge holder; and E is the elastic modulus predicted from the load–CMOD curve using Equation (8) for beams with a span/depth ratio of 4.0, where $\alpha_{0}=\left(a_{0}+H_{0}\right)/(H+H_{0})$ and $C_{i}$ is the initial compliance of the load–CMOD curve.

The critical crack length $a_{c}$ is the sum of the initial pre-cut crack length $a_{0}$ and the fictitious crack extension length $\Delta a_{c}$. $K_{Ic}^{ini}$ represents the load resistant capacity of a material before the emerging of crack propagation; and $K_{Ic}^{un}$ represents the maximum load resistant ability of a material at the critical fracture state. The difference between $K_{Ic}^{ini}$ and $K_{Ic}^{un}$ is due to the cohesive toughness $K_{Ic}^{c}$ that is caused by the energy absorbed by the cohesive force on the fictitious crack extension length $\Delta a_{c}$ progressively. A relationship between $K_{Ic}^{ini}$ and $K_{Ic}^{un}$ exists as follows:

(9) $$K_{Ic}^{ini}=K_{Ic}^{un}-K_{Ic}^{c}$$

### 5.2. Analytical Approach

The cohesive toughness $K_{Ic}^{c}$ can be also determined by analytical method [11] from the softening curve, and the detailed calculation processes are stated below.

In general, the cohesive toughness predicted by analytical method $K_{Ic}^{c,A}$ (*A* is short for analytical method) can be written as Equation (10) [11]. At the integral boundary of Equation (10), a singularity exists. The numerical results of the integration could be gained by using the Gauss–Chebyshev quadrature.

(10) $$K_{Ic}^{c,A}=\int_{a_{0}}^{a_{c}}2\sigma \left(x\right)F\left(\frac{x}{a_{c}},\frac{a_{c}}{H}\right)/\sqrt{\pi a}dx$$

where

(11) $$\begin{array}{l} F(\frac{x}{a_{c}},\frac{a_{c}}{H})=\frac{3.52(1-\frac{x}{a_{c}})}{{\left(1-\frac{a_{c}}{H}\right)}^{1.5}}-\frac{4.35-5.28\frac{x}{a_{c}}}{\left(1-\frac{a_{c}}{H}\right)}+\left[\frac{1.30-0.30{\left(\frac{x}{a_{c}}\right)}^{1.5}}{{\left(1-{\left(\frac{x}{a_{c}}\right)}^{2}\right)}^{0.5}}+0.83-1.76\frac{x}{a_{c}}\right]\\ \left[1-(1-\frac{x}{a_{c}})\frac{a_{c}}{H}\right] \end{array}$$

where σ(x) is the cohesive stress corresponding to a crack length of *x*, which can be calculated by Equation (12) when the cohesive force is linearly distributed along the fictitious fracture zone:

(12) $$\sigma \left(x\right)=\sigma \left(CTOD_{c}\right)+\left(f_{t}-\sigma \left(CTOD_{c}\right)\right)\left(x-a_{0}\right)/\left(a_{c}-a_{0}\right)$$

where $\sigma \left(CTOD_{c}\right)$ can be calculated by the bilinear softening curve as:

(13) $$\sigma \left(CTOD_{c}\right)=\sigma_{s}\left(w_{s}\right)+\frac{w_{s}-CTOD_{c}}{w_{s}}\left(f_{t}-\sigma_{s}\left(w_{s}\right)\right)$$

where *CTOD_c_* can be measured directly by using the clip gauge holder. The detailed values are listed in Table 4.

After the cohesive force σ(x) distribution along the fictitious crack zone is completely determined using Equation (12), a numerical scheme can be carried out to obtain the integral value of $K_{Ic}^{c,A}$ by using Equation (10). The bilinear softening curves obtained from inverse analysis were employed to determine the cohesive toughness $K_{Ic}^{c,A}$ of the PC and SG concrete and mortar following the above-described analytical method. The obtained analytical cohesive toughness is called $K_{Ic}^{c,A}$, and $K_{Ic}^{c,E}$ represents the cohesive toughness calculated directly from experimental results based on Equation (9).

### 5.3. Results and Discussion

#### 5.3.1. Initial and Unstable Fracture Toughness Values ($K_{Ic}^{ini}$ and $K_{Ic}^{un}$)

**Figure 5:**
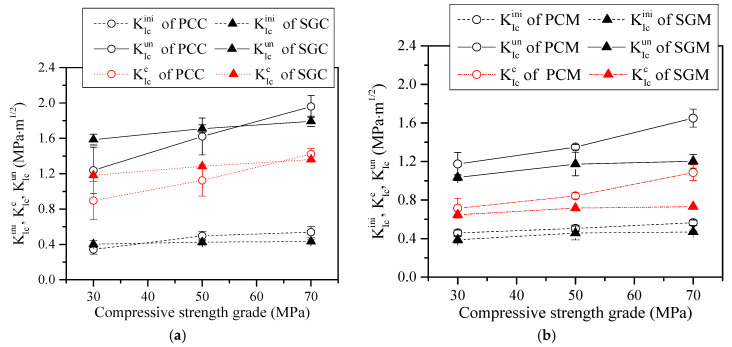
The $K_{Ic}^{ini}$, $K_{Ic}^{c}$, and $K_{Ic}^{un}$ of (**a**) PC and SG concrete and (**b**) PC and SG mortar.

Figure 5 shows the relationships between $K_{Ic}^{ini}$ and $K_{Ic}^{un}$ of the PC and SG series and the compressive strength. In general, both $K_{Ic}^{ini}$ and $K_{Ic}^{un}$ of the PC and SG series increased with the compressive strength. Figure 5a shows that the average $K_{Ic}^{ini}$ and $K_{Ic}^{un}$ of PCC increased from 0.343 MPa·m^1/2^ to 0.538 MPa·m^1/2^ (i.e., a 56.8% increase) and 1.238 MPa·m^1/2^ to 1.960 MPa·m^1/2^ (i.e., a 58.3% increase), respectively, with the increase of compressive strength from 30 MPa to 70 MPa. In the case of SGC, the increases of $K_{Ic}^{ini}$ and $K_{Ic}^{un}$ were not as significant as those of their PC counterparts; these values increased from 0.403 MPa·m^1/2^ to 0.432 MPa·m^1/2^ (i.e., a 7.2% increase) and 1.586 MPa·m^1/2^ to 1.793 MPa·m^1/2^ (i.e., a 12.6% increase), respectively (see Figure 5a). In addition, the increases of both $K_{Ic}^{ini}$ and $K_{Ic}^{un}$ of PCM and SGM with compressive strength were less significant compared with those of concrete beams (Figure 5b).

The comparison of the initial and unstable toughness values between PCC and SGC can also be seen in Figure 5a. At C30, the average unstable fracture toughness of PCC was 1.238 MPa·m^1/2^, which was 21.9% lower than that of SGC at 1.586 MPa·m^1/2^. However, with increasing compressive strength, the difference of average $K_{Ic}^{un}$ between PCC and SGC was reduced. According to Equation (4), $K_{Ic}^{un}$ is proportional to $P_{u}$, $\sqrt{a_{c}}$, and F1(acH). The relationship of $P_{u}$ between PCC and SGC with the increase of compressive strength discussed above is consistent with the trend of $K_{Ic}^{un}$. In addition, it can be found from Table 4 that the average critical crack length $a_{c}$ of SGC at C30 was 63.65 mm, which is larger than that of PCC at 60.82 mm, and such values became closer with the increase of the compressive strength for both cases. Furthermore, F1(acH) is also a monotonic increasing function of the variable acH. Hence, higher $P_{u}$ and $a_{c}$ must lead to higher $K_{Ic}^{un}$. Regarding $K_{Ic}^{ini}$, Figure 5a shows that $K_{Ic}^{ini}$ of PCC was generally slightly higher than that of SGC. Based on Equation (3), the initial cracking load $P_{\mathrm{ini}}$ is the only variable that is proportional to the initial fracture toughness $K_{Ic}^{ini}$. Referring to Table 4, the relationship of the initial cracking load $P_{\mathrm{ini}}$ between SGC and PCC is consistent with the trend of initiation fracture toughness.

It is clearly shown in Figure 5b that $K_{Ic}^{ini}$ and $K_{Ic}^{un}$ of PCM are all higher than those of SGM at all three compressive strengths. The lower initial and unstable toughness values of SGM may be due to the fact that SGM had more serious shrinkage cracks than PCM did [23,25]. Because of these existing micro-cracks, SGM featured as being more brittle and having a lower ability to resist external load. As indicated in Figure 5b, the average initial and unstable toughness values of PCM at M30 were 0.459 MPa·m^1/2^ and 1.174 MPa·m^1/2^, which were 18.3% and 11.5%, respectively, higher than those of SGM. In contrast, given the compressive strength of M70, the average initial and unstable toughness values of PCM were significantly higher than those of SGM (i.e., 20.2% and 37.3%, respectively). The aggravation of the discrepancy of the initial and unstable fracture toughness values between SGM and PCM with the increase of strength is attributed to the utilization of a higher alkali concentration activator of SGM that generated more serious shrinkage cracks [26,28], leading to a more brittle matrix and a relatively low load resistance in the case of high strength grade.

#### 5.3.2. Cohesive Fracture Toughness

Cohesive fracture toughness $K_{Ic}^{c}$ indicates the energy absorbed by the cohesive stress acting on the fictitious crack when the crack propagates stably. The comparisons of the cohesive fracture toughness between the PC and SG series are also shown in Figure 5. The variations of $K_{Ic}^{c}$ of the PC and SG series with compressive strength were similar to those of $K_{Ic}^{un}$. The higher $K_{Ic}^{c}$ of SGC as compared to that of PCC at C30 was also attributed to the denser ITZs in SGC that resulted in a higher cohesive force. On the contrary, the lower $K_{Ic}^{c}$ of SGM as compared to that of PCM was caused by more serious micro-cracks occurring in the former matrix that would reduce the cohesive stress. 

**Figure 6:**
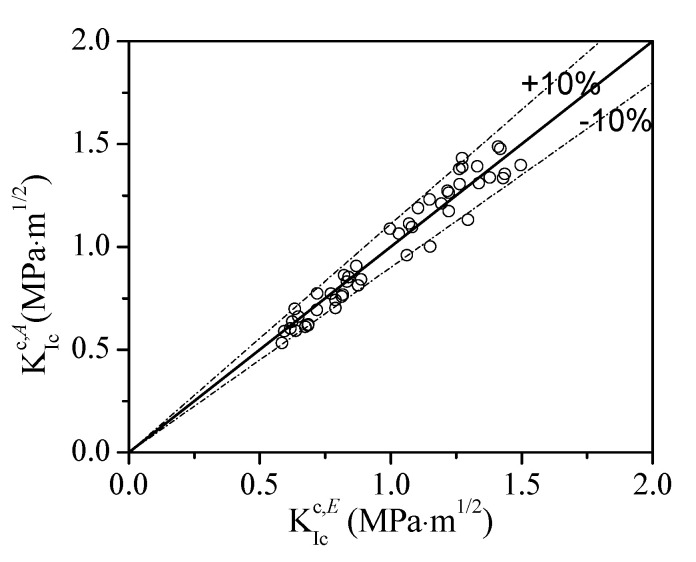
Comparison between analytical and experimental $K_{Ic}^{c}$.

Figure 6 shows the comparison between the $K_{Ic}^{c}$ evaluated by analytical method using bilinear softening curve $K_{Ic}^{c,A}$ with the experimental results $K_{Ic}^{c,E}$. It is obvious that most of the deviations between the analytical data and the experiment results are below 10%, which proves the correctness of the bilinear softening laws obtained from the backward analysis and validates the applicability of the double-*K* fracture model to SG concrete and mortar.

## 6. Conclusions

This paper determined the softening laws and fracture toughness of the PC and SG concrete and mortar by conducting TPB tests, which has been rarely studied in previous research. The softening laws obtained by inverse analysis provided critical input parameters for numerical analysis. The initial and unstable fracture toughness values of the PC and SG series were calculated referring to the double-*K* fracture model. Then, the cohesive fracture toughness of the PC and SG concrete and mortar was calculated by analytical method and experimental method, respectively. According to the results of mechanical tests and theoretical analyses, the following conclusions can be obtained.

1. For both the PC and SG series, the values of $w_{s}$ at kink point and $w_{0}$ at the stress-free point of the bilinear softening law decrease, whereas the values of $\sigma_{s}$ at the kink point generally increase with the compressive strength.
2. The first descending slopes of the normalized bilinear softening curves of PCC and SGC are generally the same, whereas PCM has a gentler first descending branch than its SGM counterpart.
3. The $K_{Ic}^{ini}$ and $K_{Ic}^{un}$ of the PC and SG concrete and mortar all increase with compressive strength increase. Moreover, both $K_{Ic}^{ini}$ and $K_{Ic}^{un}$ of SGM are lower than those of PCM given the same compressive strength.
4. The $K_{Ic}^{ini}$ of SGC is generally lower than that of PCC except for C30. Moreover, the $K_{Ic}^{un}$ of SGC at C30 is significantly higher than that of PCC and then becomes similar with increasing compressive strength.
5. The variation of $K_{Ic}^{c}$ of the PC and SG series with increasing compressive strength is similar to that of unstable fracture toughness. The $K_{Ic}^{c}$ calculated by analytical approach and experimental approach is similar, which also proves the correctness of the bilinear softening laws obtained by inverse analysis and the applicability of the double-K fracture model to SG concrete and mortar.

## Figures and Tables

**Table 1:** Chemical composition of the ground granulated blast furnace slag (GGBFS) and the Portland cement (PC) (wt %) [12].

	CaO	Al_2_O_3_	SiO_2_	SO_3_	P_2_O_5_	MgO	Na_2_O	K_2_O	TiO_2_
GGBFS	33.3	16.9	33.4	2.35	3.77	7.0	2.0	0.16	0.61
PC	64.5	5.30	21.9	2.03	–	1.51	0.19	0.62	–

**Table 2:** Mix proportions of the Portland cement mortar (PCM) and the Portland cement concrete (PCC) [12].

	Cement (kg/m^3^)	Fine Aggregate (kg/m^3^)	Coarse Aggregate (kg/m^3^)	Water (kg/m^3^)	*w/c*	SP	SR
PCM30	600	1200	–	300	0.5	–	–
PCM50	700	1155	–	245	0.35	0.09%	–
PCM70	850	1010	–	240	0.3	0.16%	–
PCC30	350	776	1164	210	0.6	–	0.4
PCC50	380	795	1192	133	0.35	0.42%	0.4
PCC70	420	782	1172	126	0.3	0.50%	0.4

Note: *w/c* is water/cement ratio.

**Table 3:** Mix proportions of the slag-based geopolymer mortar (SGM) and the slag-based geopolymer cement (SGC) [12].

	*n* (%)	*M_s_*	Slag kg/m^3^	Fine Aggregate kg/m^3^	Coarse Aggregate kg/m^3^	Water kg/m^3^	Alkali Activator		*w/b*	SR
							Sodium Silicate Solution (kg/m^3^)	Sodium Hydroxide (kg/m^3^)		
SGM30	3	1.5	783	1174	–	276	109	18	0.44	–
SGM50	4	1.5	783	1174	–	253	145	24	0.44	–
SGM70	5	1.5	783	1174	–	254	182	30	0.44	–
SGC30	3	1.5	350	746	1120	127	49	8	0.45	0.4
SGC50	4	1.5	380	724	1087	127	71	11	0.45	0.4
SGC70	4.5	2	420	694	1041	117	117	11	0.45	0.4

Note: *n* is the alkali concentration referring to the percentage of Na_2_O by mass of GGBFS, *M_s_* is modulus of the alkali activator referring to the mole ratio of SiO_2_ to Na_2_O, and *w/b* is water/slag ratio, here the total water included the water added and the water in sodium silicate solution.

**Table 4:** Average fracture parameters of the PC and SG concrete and mortar.

	*P*_u_ (kN)	*P*_ini_ (kN)	*P*_ini_/*P*_u_	CMOD_c_ (μm)	CTOD_c_ (μm)	G*_F_* (N/m)	*E* (GPa)	*a*_c_ (mm)
PCC30	2.39	1.37	57.1%	48.07	20.52	127.1	23.4	60.82
PCC50	3.24	1.99	61.2%	57.25	28.41	173.8	26.6	62.28
PCC70	3.56	2.96	83.0%	62.01	30.78	177.2	29.2	63.52
SGC30	2.99	1.54	51.6%	68.49	32.50	177.3	22.1	63.65
SGC50	3.40	1.70	49.8%	72.85	32.95	183.4	24.2	63.20
SGC70	3.43	1.73	50.3%	67.13	29.39	207.9	24.9	62.31
PCM30	1.94	1.79	92.0%	78.43	28.61	101.0	14.4	65.91
PCM50	2.21	2.02	91.5%	82.76	35.24	120.8	16.1	65.88
PCM70	2.41	2.25	93.4%	72.02	29.88	119.1	18.5	64.71
SGM30	1.79	1.55	86.4%	83.05	37.75	125.9	11.3	64.16
SGM50	2.13	1.82	85.4%	75.15	34.35	99.5	12.8	62.55
SGM70	2.14	1.88	87.7%	67.81	30.38	91.9	13.7	61.09

**Table 5:** Bilinear softening law parameters of the PC and SG concrete and mortar.

	*f*_t_	*ω*_0_	*σ_s_*	*ω_s_*		*f*_t_	*ω*_0_	*σ_s_*	*ω_s_*
PCC30	3.20	0.269	0.269	0.0468	SGC30	3.56	0.284	0.245	0.0665
PCC50	4.58	0.245	0.656	0.0378	SGC50	4.39	0.278	0.555	0.0416
PCC70	5.29	0.227	0.762	0.0290	SGC70	5.15	0.276	0.709	0.0346
PCM30	2.16	0.0754	0.513	0.0679	SGM30	2.06	0.163	0.564	0.0624
PCM50	3.40	0.0737	0.954	0.0429	SGM50	3.45	0.123	0.866	0.0213
PCM70	3.83	0.0654	0.978	0.0404	SGM70	4.27	0.111	0.960	0.0130

## Abbreviations

*a*_0_	initial crack length
*a*_c_	critical crack length
*B*	specimen thickness
*C_i_*	initial compliance of load–CMOD curve
CMOD	crack mouth opening displacement
$CMOD_{c}$	critical crack mouth opening displacement
CTOD	crack tip opening displacement
$CTOD_{c}$	critical crack tip opening displacement
*d*	mid-span deflection
E	modulus of elasticity
*f*_t_	splitting tensile strength
*H*	depth of the specimen
$H_{0}$	thickness of clip gauge holder
$K_{Ic}^{ini}$	initial fracture toughness
$K_{Ic}^{un}$	unstable fracture toughness
$K_{Ic}^{c}$	cohesive fracture toughness
$K_{Ic}^{c,E}$	cohesive fracture toughness by experiment
$K_{Ic}^{c,A}$	cohesive fracture toughness by analytical method
*P*_u_	maximum load
*P*_ini_	initial cracking load
*S*	span of the specimen
$\sigma_{s}$	cohesive stress corresponding to the kink point of bilinear softening law
σ(x)	cohesive stress corresponding to crack length *x*
$\sigma \left(CTOD_{c}\right)$	critical value of cohesive force at notch tip
$w_{s}$	crack width corresponding to the kink point of bilinear softening law
$w_{0}$	crack width corresponding to the stress-free point
